# Supplementary material for: The experiences and needs of re-entering nurses during the COVID-19 pandemic: A qualitative study
Source: Int J Nurs Stud Adv. 2021 Oct 9;3:100043. doi: 10.1016/j.ijnsa.2021.100043 (PMC8501512; doi:10.1016/j.ijnsa.2021.100043)
Supplement: Supplementary file 1 [file mmc1.docx]

**Appendix 1**

In appendix one we elaborate on the BIG-law, reserved nursing procedures and legislation regarding nursing in the Netherlands during the COVID-19 pandemic.

In the Netherlands, an existing grey area in the BIG law creates a possibility for former nurses to contribute to nursing during the COVID-19 pandemic. Registered nurses can delegate activities under specific conditions: nurses must guarantee proper supervision; be able to intervene if needed; and be able to assume that the person to whom the tasks are delegated, has the appropriate skills and knowledge (Wet Beroepen Individuele Gezondheid, 1993, §4, article 38).

Moreover, to accommodate the demand for extra qualified nurses during the pandemic within a short time-frame, the Dutch Ministry of Health made temporary adjustments in regulations ([VWS, 2020a; Bruins, 2020)](https://www.zotero.org/google-docs/?broken=grxp0T). Firstly, all BIG re-register obligations for current health professionals were suspended until further notice. Secondly, former nurses whose BIG-registration expired after 1 January 2018 are temporarily allowed to work as a nurse without the requirements to re-register ([VWS, 2020a; Bruins, 2020)](https://www.zotero.org/google-docs/?broken=grxp0T).

**References appendix 1**

Bruins, B. (17-3-2020). *Kamerbrief stand van zaken bestrijding COVID-19* [Parliamentary papers]. Retrieved from <https://www.rijksoverheid.nl/documenten/kamerstukken/2020/03/17/kamerbrief-stand-van-zaken-bestrijding-covid-19>

Ministerie van Volksgezondheid, Welzijn en Sport. (17-03-2020a) *Aanvullende maatregelen inzet voormalig-zorgpersoneel*. Retrieved from <https://www.rijksoverheid.nl/documenten/richtlijnen/2020/03/17/aanvullende-maatregelen-inzet-voormalig-zorgpersoneel>

Wet Beroepen Individuele Gezondheid BIG. (1993, November 11). Retrieved from <https://wetten.overheid.nl/BWBR0006251/2020-07-01>

**Appendix 2**

Appendix two provides the conceptual background that was used for this research. While the COVID-19 pandemic provides a unique situation, we drew inspiration from existing literature on nursing during a pandemic, and re-entering nurses, which offered relevant insights for the development of the study protocol, an interview guide and data collection.

Training

Former nurses re-entering during a pandemic need to rapidly update existing knowledge and competencies and develop new ones to become proficient in nursing during a pandemic. Research shows the need for former nurses to be aware of infection control principles and be agile in the use of personal protection equipment (PPE) [(Chan & Wong, 2007; Irvin et al., 2008; Martin, 2011; McMullan et al., 2016)](https://www.zotero.org/google-docs/?broken=GGb1S4). Nurses are the primary caregivers of vulnerable and susceptible patients and are in close contact with diseased patients. Therefore, nurses must have the knowledge to recognise and screen for possibly infected patients [(Chan & Wong, 2007; Martin, 2011)](https://www.zotero.org/google-docs/?broken=AqR4sz). Additionally, Research on re-entering nurses shows that former nurses prefer practically oriented and training focussing on competencies that are needed to work at a specific department ([Durand & Randhawa, 2002; Long & West, 2007)](https://www.zotero.org/google-docs/?broken=Qj2Qme).

Role division

Less qualified nurses rapidly re-enter care, which could create confusion about the division of roles. Existing literature on task-shifting implies that confusion on the division of roles often led to friction between healthcare workers; the hierarchy changed, since it became obscure who was responsible for what tasks [(Callaghan et al., 2010](https://www.zotero.org/google-docs/?NiW30s)). Zachariah et al. (2009) showed that not all healthcare workers felt comfortable with the additional supervisory responsibilities and the delegation of tasks due to task-shifting.

Additionally, confusion on roles may lead to quality issues. Key-findings from an evaluating study on task-shifting in Botswana revealed tasks-shifting workers often performed more activities than they were educated and commissioned for [(Ledikwe et al., 2013)](https://www.zotero.org/google-docs/?TxKvAb). A clear demarcation of responsibilities, tasks and boundaries including the advice of the involved health workers could substantiate the process of task-shifting [(Callaghan et al., 2010; Ledikwe et al., 2013; Zachariah et al., (2009)](https://www.zotero.org/google-docs/?broken=4MZNg8).

Supervision & support

Former nurses who rapidly re-entered into care with limited preparation time to master complex nursing tasks and in times of a pandemic are in need of sufficient supervision Research on the H1N1 influenza pandemic showed that junior nurses who had to rapidly skill-up in order be able to provide a high number of patients of advanced therapy, experienced feelings of anxiousness and stress due to a lack of supervision (Corley et al., 2010). Moreover, Mark and Gupta (2002), mentioned the lack of supervision as one of the main challenges for re-entering nurses. Research has observed re-entering nurses often worried about their competences or felt as if they were thrown into the deep end [(Durand & Randhawa, 2002; Mark & Gupta, 2002)](https://www.zotero.org/google-docs/?XOVSpQ)

Mental health needs

Several studies have postulated about the adverse psychological effects of nursing during the COVID-19 pandemic [(Kang et al., 2020; Lai et al., 2020;](https://www.zotero.org/google-docs/?ZbjPnJ) Zhu & Xu [et al., 2020)](https://www.zotero.org/google-docs/?ZbjPnJ). Research indicates that nurses responding to the COVID-19 outbreak in Wuhan China often experienced anxiety and stress, which was associated with the long working hours, extra work pressure, close contact with infected patients and the lack of PPE [(Lai et al., 2020;](https://www.zotero.org/google-docs/?UyKE0n) Zhu & Xu [et al., 2020](https://www.zotero.org/google-docs/?ZbjPnJ)[)](https://www.zotero.org/google-docs/?UyKE0n). A key factor contributing to fear amongst nurses was spreading of the disease amongst colleagues and family members and the increasing number of deaths [(Lai et al., 2020;](https://www.zotero.org/google-docs/?DO0uH9) Zhu & Xu [et al., 2020](https://www.zotero.org/google-docs/?ZbjPnJ)[)](https://www.zotero.org/google-docs/?DO0uH9).

Private life

Providing care during a pandemic could have a demanding influence on the personal life of former nurses. Existing research on the preparedness for an influenza pandemic recognised worries amongst nurses about shortages in staff, leading to a demand to work extra shifts and longer hours [(McMullan et al., 2016)](https://www.zotero.org/google-docs/?broken=KINDPT). Moreover, research showed that nurses worried about exposing their environment to an increased risk of infection (McMullan et al.,2016; [Corley et al., 2010)](https://www.zotero.org/google-docs/?Sy7pdW). In two additional analyses of the willingness to work during a pandemic in America, nurses mentioned similar worries [(Irvin et al., 2008; Martin, 2011)](https://www.zotero.org/google-docs/?rW3GEE). As a solution, McMullan et al. (2016) noted the importance of available resources and information to reassure nurses and their families.

**References appendix 2**

Callaghan, M., Ford, N., & Schneider, H. (2010). A systematic review of task-shifting for HIV treatment and care in Africa. *Human Resources for Health*, *8*(1), 8.<https://doi.org/10.1186/1478-4491-8-8>

Chan, W. F., & Wong, T. K. (2007). Preparing for pandemic influenza: Revisit the basics. *Journal of Clinical Nursing*, *16*(10), 1858–1864.<https://doi.org/10.1111/j.1365-2702.2007.01831.x>

Corley, A., Hammond, N. E., & Fraser, J. F. (2010). The experiences of health care workers employed in an Australian intensive care unit during the H1N1 Influenza pandemic of 2009: A phenomenological study. *International Journal of Nursing Studies*, 47(5), 577–585.<https://doi.org/10.1016/j.ijnurstu.2009.11.01>

Durand, M. A., & Randhawa, G. (2002). Nurses’ views about returning to practice after a career break. *British Journal of Nursing*, 11(7), 477–485. <https://doi.org/10.12968/bjon.2002.11.7.10147>

Irvin, C. B., Cindrich, L., Patterson, W., & Southall, A. (2008). Survey of Hospital Healthcare Personnel Response during a Potential Avian Influenza Pandemic: Will They Come to Work? *Prehospital and Disaster Medicine*, *23*(4), 328–335.<https://doi.org/10.1017/S1049023X00005963>

Kang, L., Ma, S., Chen, M., Yang, J., Wang, Y., Li, R., Yao, L., Bai, H., Cai, Z., Xiang Yang, B., Hu, S., Zhang, K., Wang, G., Ma, C., & Liu, Z. (2020). Impact on mental health and perceptions of psychological care among medical and nursing staff in Wuhan during the 2019 novel coronavirus disease outbreak: A cross-sectional study. *Brain, Behavior, and Immunity*.<https://doi.org/10.1016/j.bbi.2020.03.028>

Lai, J., Ma, S., Wang, Y., Cai, Z., Hu, J., Wei, N., Wu, J., Du, H., Chen, T., Li, R., Tan, H., Kang, L., Yao, L., Huang, M., Wang, H., Wang, G., Liu, Z., & Hu, S. (2020). Factors Associated With Mental Health Outcomes Among Health Care Workers Exposed to Coronavirus Disease 2019. *JAMA Network Open*, *3*(3), e203976–e203976.<https://doi.org/10.1001/jamanetworkopen.2020.3976>

Ledikwe, J. H., Kejelepula, M., Maupo, K., Sebetso, S., Thekiso, M., Smith, M., Mbayi, B., Houghton, N., Thankane, K., O’Malley, G., & Semo, B. (2013). Evaluation of a Well-Established Task-Shifting Initiative: The Lay Counselor Cadre in Botswana. *PLoS ONE*, *8*(4).<https://doi.org/10.1371/journal.pone.0061601>

Long, J., & West, S. (2007). Returning to nursing after a career break: Elements of successful re-entry. *Australian Journal of Advanced Nursing*, *25*(1), 49-55.

Mark, S., & Gupta, J. (2002). Reentry Into Clinical Practice: Challenges and Strategies. *JAMA*, *288*(9), 1091–1096.[https://doi.org/10.1001/jama.288.9.1091\](https://doi.org/10.1001/jama.288.9.1091%5C)

Martin, S. D. (2011). Nurses’ ability and willingness to work during pandemic flu. *Journal of Nursing Management*, *19*(1), 98–108.<https://doi.org/10.1111/j.1365-2834.2010.01190.x>

McMullan, C., Brown, G. D., & O’Sullivan, D. (2016). Preparing to respond: Irish nurses’ perceptions of preparedness for an influenza pandemic. *International Emergency Nursing*, *26*, 3–7.<https://doi.org/10.1016/j.ienj.2015.10.004>

Ministerie van Volksgezondheid, Welzijn en Sport. (2020b). *Beoordelingskader verpleegkundigen*. Retrieved from <https://www.bigregister.nl/herregistratie/documenten/publicaties/2017/03/03/beoordelingskader-verpleegkundigen>

Zachariah, R., Ford, N., Philips, M., S.Lynch, Massaquoi, M., Janssens, V., & Harries, A. D. (2009). Task shifting in HIV/AIDS: Opportunities, challenges and proposed actions for sub-Saharan Africa. Transactions of the Royal Society of Tropical Medicine and Hygiene, 103(6), 549–558. <https://doi.org/10.1016/j.trstmh.2008.09.019>

Zhu, Z., Xu, S., Wang, H., Liu, Z., Wu, J., Li, G., Miao, J., Zhang, C., Yang, Y., Sun, W., Zhu, S., Fan, Y., Hu, J., Liu, J., & Wang, W. (2020). COVID-19 in Wuhan: Immediate Psychological Impact on 5062 Health Workers [Preprint]. Psychiatry and Clinical Psychology.<https://doi.org/10.1101/2020.02.20.20025338>

**Appendix 3**

Appendix three contains the interview guide that was used during data collection.

The following questions were asked during the recruitment of participants to assure a varying sample of former nurses working in different settings.

- What is your age?
- What is your profession?
- How many years of experience do you have as a nurse?
- In which department(s) and in what kind of institution(s) (hospital, nursing home, home care etc.) did you work?
- Did you follow any nursing specialisations? If so: what is your nursing specialty?
- In what year did you stop working as a nurse?
- Were you BIG registered at the time of your re-entry into nursing?
- In which department and in what kind of institution are you working momentarily as a nurse?
- How long have you been back in nursing practise again?

| Introduction |
| --- |

| Intro | - Introducing researcher. - Thankyou for participating. - Are there any questions regarding the information letter you received? |
| --- | --- |
| Purpose of the study | - The goal of this interview is to gain more insight into your experiences as a re-entering nurse during the COVID-19 pandemic in the Netherlands. - Through this research we hope to identify what former nurses need to help them process their re-entry during a pandemic as smoothly as possible. - Therefore, I would like to ask you to share your experiences and perspective as openly as possible. |
| confidentiality | - Informed consent. - This interview will be anonymous, as was explained in the information letter. This means that personal information will not be mentioned in the rapport. Moreover, information will not be shared by others who were not involved in this research process. - The information that you provide us with will only be used for the purpose of this study. - As mentioned in the information letter and the informed consent, I would like to audiotape our conversation. Therefore, according to the regulations, I would like to ask you again for your permission to audiotape our conversation. - It is your right to stop the interview and retract yourself from the study at any time. |
| Interview structure | - The interview will take about +/- 90 minutes - Structure of interview: - First, I would like to address your background, Second I would like to talk about what happened before you returned into nursing. Lastly, I would like to walk through your re-entering process from the first day until now. |

| Themes |
| --- |

| **Background**   - First I would like to walk through the questions you answered prior to this interview. - Why did you decide to quit nursing in the past? - Why did you decide to re-enter as a nurse? - How was your re-entering process organised? - Where did your re-entering process take place? | **Sub-questions/topics for probing**   - Study, work, specialisations. - What factors contribute to this decision? What kind of considerations did you make? - How long did it take before you re-entered? - Can you tell me a bit more about the department? |
| --- | --- |
| **Training (BIG registration)**     - Did you participate in any form of training before you re-entered? - What was the focus of the training program? - How did you experience this training? - Did you feel well prepared for your return? - What would an effective training programme for re-entering nurses in your situation look like? | - If yes: How was this organised? Who initiated this training? If no: why not? - Practical/theoretical. Focus on pandemic? - Difficulty/Usefulness/Clarity (both positive and negative) |
| **Back in practise**   - Could you tell me how your first day back in practise went? - What struggles did you encounter on your first day? - What went well? - How did COVID-19 influence daily working practises? | - How was this organised? How did you experience this? What was your department like? How did COVID-19 influence this? - How did you deal with this? |
| **Rolverdeling**   - What is your role as a returning nurse (fully independent)? - How do you experience your role? - How does the COVID-2019 pandemic affect the division of roles in your department? - Are colleagues aware of your role in the department? | - Are there clear agreements regarding your role and responsibilities in practice? Could you give examples of what you can and cannot do? How are these boundaries determined? - Do you feel sufficient competence? Are you experiencing any problems with your role? If answered yes; how do you deal with this? - What does this mean for your re-entering process? - How do they deal with this? |
| **Team**   - What is the team in which you work now like? - How does your new team contribute to your re-entry? - How does the COVID-19 pandemic affect the social dynamics in your new team? | - How do colleagues approach you? How do your new colleagues react to your re-entry? |
| **Supervision and guidance**   - How is supervision arranged during your re-entry? - How do you experience the degree of supervision and support during your re-entering process - How is new information and / or the use of new measures related to COVID-19 communicated to you? | - Who can you turn to for advice and guidance? - What went well and what could have gone better? How does this affect your re-entry? - How do you experience this? |
| **Work-life balance**   - How did your family/environment react to your return? - How does your re-entry affect your private life? - How do you experience working irregular hours - How is the current COVID-2019 pandemic affecting this? | - How do you handle this? - How do you handle this? - How do you handle this? |
| **Mental health**   - How does re-entry affect your mental health? - How do you experience the current workload? - What attention does the organization / department give to the maintenance of mental health? | - How do you handle this? What is the influence of COVID-19 on this? - How do you handle this? - How does this affect you? What do you think their role should be in this? |
| **Changed profession**   - Has much changed in nursing since you left? - How is the COVID-2019 pandemic affecting this? - How does your organization and department deal with the changes you experience? | - Could you give examples? How do you experience this? How do you deal with this? - What should the role of the organization / department be in this? |
| **Integration into practise**   - Looking back, what has changed since your first day in practice? - What are the bottlenecks you are currently facing compared to your first day? - Which uncertainties do you still face compared to your first day? - Are you thinking about continuing working as a nurse even after the pandemic is over? - What should change in healthcare to attract more former nurses? - What advice would you like to give to new returning nurses? | - What is going better? - Has a lot changed? How do you deal with this? What concrete steps should be taken to resolve these bottlenecks? - Has a lot changed? How do you deal with this? What concrete steps should be taken to resolve these insecurities? - Why yes/no? - Why yes/no? |
| **Ending**   - Are there any important topics / points that we have not yet discussed and which you would like to address? - Thanks for participating. - Could I approach you again if new questions arise? |  |

**Appendix 4**

Appendix four contains the coding scheme that was used to analyse data, categorised on themes, subcodes and codes.

| **Themes**  Subcodes  Codes | **Description** | **Example** |
| --- | --- | --- |

| **Theme: Job description and scope of practise** |  |  |
| --- | --- | --- |
|  |  |  |
| Executing technical nursing activities. | Segment in which returners discuss the agreements about taking up technical nursing activities. Including reserved actions. | *“There was someone who had wounds on her heels that had to be treated, but I was with someone who was from the nurse aide staff. He said, ‘I can't do that, someone from the level of nurse assistant staff should do that, he has to take care of those wounds.’ Yet that person said, ‘you are a nurse, if I tell you how to do it, can you do it?’ I thought yes, this can't go wrong. It was nothing with injections or with drugs. I have been taking care of wounds for so long. So then I said, ‘I will do it , but you should check it afterwards.’ Well, I am competent, but actually I am not qualified. ”* |
|  |  |  |
| Clarity about role within the team | Segments in which returners discuss whether their role was clear within their team or not. Including the expectations of the team members towards the returners. | *“Well, I got a call on a Tuesday asking if I wanted to come and work and if I could come within 48 hours. So I came there and their intention was that they immediately needed people who knew the ropes and could start working. While I came in with the idea, guys, I haven't done anything in healthcare for twenty years, help. I really want to do something, but you have to say what the intention is and just give me instructions. “* |
|  |  |  |
| Scope of practise | Contains codes related to how the returners' role is ultimately executed regardless of their job description and how they experience this. | - |
| Comfortable / competent in role | Contains segments in which returners indicate that they felt competent in their role or to perform certain actions. | *“Well, I still feel like I have picked up things very quickly and could get started, I thought that was a very nice feeling. “* |
| Supporting role | Contains segments in which returners indicate that they perform a supporting role (regardless of the position in which they were previously hired). For example, some former nurses are hired as an independent nurse but ultimately perform in a more supportive role. Including reasons and experiences. | *“It was true that the nurses were ultimately responsible, but fortunately I never was alone on the ward after the first shift. So I was always with a nurse with a lot more experience. She was ultimately responsible, so I kept myself in the background a bit. ”* |
| Defining boundaries | Segments in which returners indicate that they have to convey their own limitations regarding their competences | *“Uhm protect your boundaries, so know what belongs to your responsibility. And see if that is clear for the team, because otherwise you start with skewed expectations. “* |
| Logistical tasks | Segments in which returners indicate that (mainly in the beginning) they were also busy with logistics tasks due to the establishment of new departments. For example arranging the department, ordering missing materials, etc. | *“It was like this the first day for everyone, because what do we actually have? do we have a first aid kit, do we have an IV bag. You're trying to organize it. You try to supply the department and do all the logistics as well as possible.* |
| Grow in position | Segment in which returners indicate how they grow in their role as their re-entry process progresses. Including segments in which returners indicate that growth remains limited. | *“In the beginning it was a bit uncomfortable because I have never done such physical work in a nursing home with people, and I did notice that I became a bit more comfortable with that towards the end. So that I just learned to really deliver physical care like that, so I learned from it. I am more skilled now than then. ”* |
| Proactive | Contains segments in which returners indicate that they should be proactive during the re-entry process. For example, when taking on tasks and asking questions to become more knowledgeable. | *“Everyone is busy with things that need to be arranged. Clinics have to open again, how should patient flow be organised, and you have a clinic that has to be moved outside, so they are now much more concerned with that. So if you end up in such a crisis situation and you are going to re-enter then you also need to have a proactive attitude and be capable of finding your own way around all this."* |
| Independent nurse | Segments in which returners report that they function as an independent nurse (regardless of the role in which returners were previously hired). Eg hired as a buddy, but eventually started working as an independent nurse. Including reasons and experiences. | *“That they said within a day ‘(participants name), maybe you can just go back to work as an IC nurse. That might be a bit more convenient than as Buddy’. The next day I actually just started as an IC nurse. ”* |
| Insecurities in role | Segments in which returners discuss insecurities within their role. | *“Yes I also felt a bit clumsy or something. I mean you have to adjust again. The last time I really washed someone's buttocks was in the nineties, so you know. So I thought... I felt a bit too .. are they really happy with that? Can I add value?”* |
| Nervousness about the unknown | Segments in which returners say that they find the new aspects of the department and an unknown illness particularly tense. | *“Yes, it was very tense for everyone, it was the first week since the department was established and everyone started working there, so it was new for everyone and everyone was a bit tense, because it was corona after all . “* |
| Tasks picked up quickly | Segments in which returners discuss the tasks they picked up quickly/easily. | *“Yes, that's especially when you're standing with the patient and you have to take care of the patient and you have to help the patient wash. You see how every nurse starts washing, and running, and communicating with the patients, and changing the beds. Those are actions you have done so many times and the observations you make, and the conversations you have at the bedside. Yes, you just pick these tasks up again and then you think, yes, fortunately not much has changed. “* |
| Affinity with role | Segments in which returners express an affinity with caring and their role and are satisfied with their role. For example, working in healthcare again feels familiar. | *“As very positive and familiar. That sounds very strange, but I thought ‘oh dear, after 28 years suddenly I get back to such an organization and at the bedside. That is*  *of course the reason why I once did my training. I really like that feeling; once a nurse always a nurse ”* |
| Time / space to learn within the role | Segments showing that returners needed a little more time to master certain tasks and/or realize that they cannot learn everything at once. | *I also noticed that when I walked through the corridors - well I have a fast pace anyway - but that*  *the nurse said just walk a bit at a slower pace. Yes, you have to give yourself time*  *to get the hang of it again.* |
| Open attitude towards role | Segments showing that returners had an open and flexible attitude when they re-entered into care and had few expectations or requirements for their role. "Just wanted to help" | *“Yes, and not anxious or anything. There just wasn't enough time to study everything very well. So, it was just like, ‘go for it’ and hope that I can patch it up if I misjudge it once. Yes, I can do that anyway, but you also have people who are very concerned about that. to do something wrong. I really thought, ‘I will just do it and I know what I can do and I know what my limitations are’. That's how I work.”* |

| **Thema: Mental health** |  |  |
| --- | --- | --- |
| Mental health impact | Contains codes with segments about the impact of the re-entering process on the mental health of the returners. | - |
| Coping with death | Contains segments in which returners talk about the emotion of patients dying about corona or fear of dying of patients. | *“Yes, what I found very intense was that the manager also said, ‘yes gosh, Corona is a serious disease, because the situation of patients can deteriorate very suddenly and they can even die, which seems to be a very intense image’. So I was very afraid of that. Like oh dear, if that happens I might have that image of how someone literally suffocates in his own fluids on my mind for the rest of my life. ”* |
| Working with PPE | Segments in which returners say that working with PPE was demanding. | *“But it was continuously dressing and undressing, because for every room in and out you have to get your clothes on and off every time, so we were all the time getting dressed and undressed. That was of course very labor-intensive and we used a lot of material. But it worked well, there was still enough material, so it was just a lot and very quickly responding to that, "* |
| Fear for infection | Segments in which returners talk about the fear of becoming infected themselves or the fear of infecting their family. | *“You know, when I got home I took a shower and only then I felt like I was really virus-free. I thought that was quite scary.* |
| Difficulties with consequences for patients' families | Segments in which returners indicate that they found it difficult that family was not allowed to visit the patient. | *“But you can get a little angry or frustrated yourself, that you think ‘yes she will die soon and then their family just has not seen your mother again, is it really worth it. I found it very difficult, "* |
| Intensive | Segments in which returners indicate that their re-entering process was experienced as an intensive period. | *“Physical but also mentally, all the impressions... one day is easier than the other.. Yes, there are things that stick with you, like what you go through with people, families and the amount of information. You try to look things up yourself in the evening. So for me yes, but not too much, no not too much. It did not limit me. ”* |
| Letting go | Segments in which returners say it is important to let go of work. | *“I actually noticed that I can let go of this work more easily than when I'm still busy with all those managerial duties. I take that work home with me more often than the caregiving.* |
| Positive influence on mental health | Segments in which returners explain that the impact of their re-entering process on their mental health was actually positive or was not influenced at all. | *“Well not actually, well positive. Not negative anyway. It made me happy to be able to work again. Yes, you know I gained a valuable experience. I worked as a nurse again. but I did not impair my mental health.* |
|  |  |  |
| Mental health support | Codes with segments explaining the mental health support provided by the organizations and other support / coping mechanisms that re-entering nurses experienced / used. | *-* |
| Acknowledgement from environment | Segments in which returners speak about the acknowledgement and appreciation they have received from their environment and the organization in which they have worked. | *“In (name city) you can nominate other people for a price, people who have done something good for the city. I got chocolates last week, which are chocolates in the shape of a (characteristic of the city). I received that and we took a photo, which was posted on Facebook.* |
| Mental health support from manager | Segments in which returners report that team leaders / managers are vigilant about the mental health of employees | *“And then I thought maybe this is the time to say, ‘I have to take care of myself and I will go home to sleep’ and then a few days later I got a call from the team leader asking, ‘how are you and what happened’. Ehm, so they really pay attention to each other.”* |
| Media training | Segments about a media training that was set up by the business support team and its usefulness | *“Um well, I must say at first I was uncertain about how it would affect me. And because of the support that you get and the explanations on how things go, I only became less insecure about it and I actually felt supported in the choices that I made. And that just helps me in daily life, because I know how to reduce the news and how to deal with everything that appears in the media and all the uncertainty. ”* |
| Psychological help | Segments in which returners say that there was a possibility to have a conversation with a psychologist or coach. | *“And I must also be honest, at (name of hospital) - and that is the case with many hospitals- it is all very well organised. Our psychologists said, ‘we are also available for our own employees’, and there was a consultation hour or people could call during the night. So people were really facilitated in this.”* |
| Talking with colleagues or family | Segments in which returners report that they sought support from colleagues and family. | *"Also the contact with colleagues. You have a number of colleagues with whom you just notice that you can get along well, and um, you can talk to them."* |

| **Theme: guidance and supervision** | *Theme about how guidance and supervision is arranged and how returning nurses experience this.* | - |
| --- | --- | --- |
| First point of contact / mentor | Segments from which it emerges whether returners are linked to a permanent mentor or / and that a first point of contact has been assigned. Including experiences and wishes. | *"Uhm and in practice I think it would be very good if someone was linked to a permanent mentor."* |
| Evaluation moment | Segments showing that returners make use of moments to evaluate or whether they wish to do so. | *“Yes, that from time to time they have a conversation with that returner,*  *like 'gosh what problems do you actually encounter, what could we do differently and what could be improved?’ ”* |
| Seeking guidance | Segments showing that returners themselves seek guidance and arrange supervision. | *“ In the beginning I was really linked to someone, when I started caring for patients independently, I always made sure that I had someone to go to, for myself, I arranged that myself. “* |
| Supporting role of manager | Segments in which returners speak about the manager's supporting role in the department. | *“Anyway, that's really ten points for our team management, because that's real. I do not know; no service is too crazy, you can request anything and they just see what they can do for you.“* |

| **Thema: training** |  |  |
| --- | --- | --- |
| *Received training* | *Codes of segments related to forms of training that returners have followed before or during their re-entry, including their experiences with this.* | - |
| Department-specific instructions | Contains segments in which returners talk about the instructions / introduction they have received about the department where they are placed. | *"What was expected of us, the rules, and someone came from the fire prevention department and from the GGD. So it was actually half a day. They showed us around, arranging uniforms, and everything."* |
| Practical training / Skills lab training | Contains segments in which returners talk about the practical training they have had. For example, a skills lab. | *“That was really very well organized together with the UT and the skills lab here in the hospital. Uhm, they started training people, so again the infusion pumps and learning things like that again. ”* |
| COVID-19 specific training | Contains segments in which returners talk about the instructions / training they have had focused on COVID-19. | *No, but we had very good instructions about that. Ehm, the red cross had arranged that for us. There were pamphlets hanging everywhere about the order in which you should take things off. And that nurse teacher, while we were busy with that briefing, she also gave good instruction. So I had that in mind, I knew that.”* |
| Self training | Contains segments in which returners report that they have sought out (extra) training themselves. | *“There is an e-learning that I just do and that helps enormously and that provides me support. I also check that COVID-19 site every day, because I think*  *It's really great that they have also posted things about how to stay unharmed as a nurse. but where can you find protocols and*  *e-learnings and things like that.”* |
| E-learnings | Contains segments in which returners talk about the e-learnings they have followed. | *“There are several modules about for example resuscitation but also blood care. The organization has some kind of a learning environment where you are guided through the material and can do a practice test and eventually you can pass that thing"* |
|  |  |  |
| No (full training) training | Codes with segments showing that returners say that they have not received (full) training. |  |
| No training due to low complex care | Segments in which returners indicate that training was not necessary or not provided due to the low complexity of the tasks aligned with their position. | “*Uhm, I did not experience it as something I missed, uhm absolutely not, because it is just like you know, like cycling, knowledge on basic care is still there. I can do that ”* |
| No time for full training program | Segments in which returners indicate that there was no time to do a training before their return. | *“Actually, from the point of view of my nursing background, I think that there should be something, but we are now in a hectic situation in which I just see that a lot of people who work in practice are doing their utmost best. They actually also think that they should support more. "* |
|  |  |  |
| Training needs |  |  |
| Individual training programme | Segments in which returners discuss the importance of individualized training | *“Returners are people who often have quite some life-experience. It would be nice if training relates to this, so they do not have to do everything by default, but where you look at what someone needs. I have been out of the running for 28 years, but someone who was only absent for five years needs something else entirely.”* |
| Practicing transfers/lifts | Segments in which returners indicate that they want to receive training on patient-lifts and transfers | *Uhm in this department - now again, I come back to that stupid patient lift (laughter) - I would have liked it if I would have known that from the beginning. I would have been able to work more independently and much faster”* |
| Practical training (need) | Segments in which returners express their desire for practical training. Including which practical actions they want to learn, e.g. reserved actions, pumps, etc. | *“For example, in terms of my reserved actions, I would have liked it if the things that had been changed or were adjusted in the protocol.. that someone would have demonstrated how to do those things right. Maybe I would have liked a skills lab, but it was not possible. ”* |
| COVID-19 related training (need) | Segments in which returners indicate that they would have liked certain corona-related training. | *"I would have wanted to know especially a bit more about administering oxygen or also about the clinical picture itself. Um, I might have wanted some more information about this."* |
| EPD training | Segments in which returners indicate that they would have liked to receive more training about the electronic patient file. | *“We should have invested a lot more time in that. We should have practiced with each other. We only went through some buttons on a powerpoint, like here you have the client, here you have the file, here you see this and here you see that. Well, of course that's not okay”* |
| Learning changed protocols /treatments | Segments showing that returners would have liked to receive some more guidance on changes in protocols and treatments. | *“Maybe discuss with someone which protocols are very common. For example, I had a patient with hyperglycemia and I treated him in the way I thought I still had to, but the protocol had just been adjusted slightly. Anyway, if you are already on the work floor the next day, you do not have time to read 200 protocols. That just won't work. “* |

| **Theme: positive team dynamics** |  |  |
| --- | --- | --- |
| Positive team dynamic |  |  |
| Team culture not yet established | Segments that show that there is no established team culture yet. Dynamics within the team have not yet been determined | *“So that makes it very different, everyone is a bit scanning, who are you, what can you do and what do you want. That is just completely different from entering an established group. I think if we could have kept this up for a few more months, you would get that kind of dynamic, but it was not there yet.”* |
| Team needs each other | Segments in which returners indicate that they had to work together because of the pressure of the crisis. | *"The team spirit, it was just almost fun. Just everyone was like ‘maybe it will take a while and we just need you’ and we needed them."* |
| Solidarity within the team | Segments showing that returners experience the team as willing to help. Solidarity towards each other. | *“Yes, everyone helps each other where they can. Everyone also puts the client first. I liked that so much, there were no hierarchical things or anything, everyone just did what they could, and everyone helped each other.”* |
| Appreciation from the team | Segments in which returners indicate that they feel appreciation from their team | *“They were actually very happy with everything you could do and they really emphasized that. I found that so surprising. Yes, I admitted that, it really felt very welcome ”* |
| Combatant team | Segments where returners indicate that the team is determined and combative to tackle the problems surrounding the pandemic together as a team. | *Ehm what I think is cool, that despite the whole new situation, despite all the hectic, I thought it was cool to discover that everyone had an enormous drive to get it all done, to get that job done. So everyone was very combative and all wanted to get involved.”* |
| Unity | Segments where returners indicate that they feel a great sense of unity within the team. Including wording such as: being part of the team, faces are in the same direction, close team, openness, etc. | *“And so a culture of cooperation arose, and suddenly there was no hierarchy anymore. People just started to consult with each other. There were things adapted in practice. People were open to any comments. ”* |
|  |  |  |
| Uninvolved in team | Segments where returners describe moments when they felt less involved in the team. | *For example, a colleague said, ‘I am fine with taking care of that patient, but then I do want a good buddy.’ The room was filled with all the buddies, so when they picked someone, you knew that was a good buddy, and the rest was rubbish. You have to imagine that you are completely dressed up with a mask and eye protection, and then that is being said. I could not comprehend it.”* |
|  |  |  |
| Varying team | Codes with segments indicating that returners worked with a varying team and the consequences thereof | *-* |
| Estimating team | Segments showing that returners and their colleagues had difficulty estimating what they could benefit from each other. For instance, in level of knowledge. | *But it still took a while, because every time I was in a department with other colleagues, and everyone had a different background. So, we had to see who did what, but everyone was also very honest about that ‘I dare to do this, or I do not dare to do that, or I have not done that yet.’ Together we managed.* |
| New and unknown backgrounds | Segments showing that returners worked together with different colleagues from different backgrounds. | *“I also have to say that a number of those people are said secondment workers. So a department consists of only twenty permanent employees, such a small department the (name of hospital) has. Yes, the rest that was flown in from other departments or were secondment workers. “* |

**Appendix 5**

We focus on the experiences and needs of re-entering nurses in the context of the COVID pandemic, recognising this as an opportunity to increase the workforce during times of crisis. We did not specifically focus on the retention of these nurses after the crisis or on the specific reasons why participants initially decided to leave nursing. This is beyond the scope of our study and research question and was therefore not included in the body of our manuscript. However, we are aware that knowledge on these topics provides information on issues needed to address to create a sustainable workforce of re-entering nurses, potentially contributing to the diminishment of chronic shortages. We do provide a short overview of why nurses initially decided to leave nursing, why they returned during the pandemic and if they were thinking of continuing working as nurses after the crisis within this appendix.

*Reasons to leave nursing*

Most nurses mentioned having a combination of multiple reasons that had led to their resignation. Participants often explained having a specific new interest or ambition that motivated them to explore other career options. For instance, five participants mentioned their wish to influence the organisation and quality of healthcare by stepping into management or policy positions. Four participants mentioned a lack of challenge within their nursing jobs. Others mentioned the desire to pursue a different study or having an interest in other facets of healthcare. Moreover, six participants explained struggling with combining work and caretaking of children. Especially, working irregular hours and weekends, caused difficulties for some to arrange fitting day-care for their children. Furthermore, six participants mentioned that they struggled to endure irregular shifts such as night-shifts, hence leaving nursing resulted in more regularity. Three participants mentioned an increased workload as a reason to change careers, and two participants said that organisational changes and financial cuts led to their resignation. Another four participants had an additional personal reason to leave nursing.

*Reasons to re-enter into nursing during the pandemic.*

Participants unanimously explained that the eagerness to help in dire times of the pandemic was one of the main reasons to return. Nearly all participants mentioned that they would not have re-entered nursing at this moment if the COVID-19 pandemic had not emerged. In addition to the willingness of participants to help in times of a pandemic, two other factors facilitated the opportunity to (temporarily) return into nursing. Firstly, seven participants explained that COVID-19 reduced the available amount of work in their current jobs. Secondly, two participants reported being currently in-between jobs.

*“ I thought, I can not permit myself to stay and sit comfortably behind my laptop and think ‘ yes, have fun colleagues’ because they will always feel like some sort of colleagues.” (Participant at COVID-19 department in a nursing home setting) R9*

Nearly all participants intended to re-enter temporarily. One participant decided to resign from one’s current job and completely re-enter as a nurse, while another former nurse considered combining one’s current job with an occasional nursing shift. Moreover, one participant enrolled in a management position at the department where this participant was employed as a former nurse and intended to continue working as a nurse once a month to maintain a connection with the workplace.
