# Supplementary material for: The experiences and needs of re-entering nurses during the COVID-19 pandemic: A qualitative study
Source: Int J Nurs Stud Adv. 2021 Oct 9;3:100043. doi: 10.1016/j.ijnsa.2021.100043 (PMC8501512; doi:10.1016/j.ijnsa.2021.100043)
Supplement: Supplementary file 2 [file mmc2.docx]

**AUTHOR CHECKLIST**

| ***PART 1 Basic requirements - For the items below, please tick or the relevant page number in the right hand column to confirm you have included/addressed the items in your manuscript. For more detail please consult the guide for authors:*** | | Insert a tick or page number(s) |
| --- | --- | --- |
| Ethical approval and informed consent | For all research papers *only*, please ensure that your manuscript includes details of the ethical approval granted including the body that granted it and any reference number. If ethical approval was not required, give a clear statement of the basis on which this assessment was made, with reference to the ICMJE requirements. This should include confirmation of informed consent by participants. Place this **at the end of you methods section.** | p.10 |
| Study registration | Give any study registration number (e.g. ISRCTN) in the **abstract and in the body** of the paper. For clinical trials (as defined by the ICMJE), the abstract should include the registration date and the date of first recruitment. [not applicable to letters / editorials] | N.A |
| Funding sources | State sources of funding and the role of funders in the conduct of the research or include a statement ‘no external funding’ **at the end of the paper**. | p.29 |
| Conflict of interests | State any actual or potential conflicts of interest in a section **at the end of the paper**. If there are none, include a statement “Conflicts of interest: none”. The substance of this declaration should match details provided in file(s) uploaded at submission. | p.27 |
| Title | The title is in the format ‘Topic / question: design/type of paper’ [not applicable to letters / editorials] | Title page |
| Abstract | A **structured** abstract of no more than 400 words appropriate to the design of the study (and as directed by relevant reporting guidelines) is included **at the beginning** of your paper. No references are cited in the abstract. [not applicable to letters / editorials] | p.2-3 |
|  | You may include a final section to their structured abstract with an additional sinal section: “Tweetable abstract” summarising a key message in no more than 140 characters. [not applicable to letters / editorials] | p.3 |
|  | No abbreviations (other than SI units) or references are to be used in the title or the abstract of the paper | Done |
| Key words | Give between four and ten key words, which accurately identify the paper's subject, purpose, method and focus. Use the Medical Subject Headings (MeSH®) thesaurus or Cumulative Index to Nursing and Allied Health (CINAHL) headings where possible (see <http://www.nlm.nih.gov/mesh/meshhome.html>). | p.3 |
| Contribution of the Paper statements | **After the abstract** under the headings "What is already known about the  topic?" and "What this paper adds" give 2-3 single sentence bullet points (each) summarising key contributions. [not applicable to letters / editorials] | p.4 |
| Abbreviations | The paper does not contain any abbreviations, acronyms or “initialisms” other than the limited exceptions noted in the guide for authors. | Done |
| Other Published accounts | Other published and in press accounts of the study from which data in this paper originate are referred to in the paper and the relationship between this and other publications from the same study is made clear in the paper. [not applicable to editorials or letters unless reporting analysis / data] | See below |
| ***Please provide below full references to ALL other publications from this study and explain the relationship to the current paper. To assist editors upload copies of papers where the abstract / full text is not readily available (including those under review elsewhere, which will be treated in strict confidence).***  Our manuscript was recently submitted to the preprint servers of Medarxiv.  <https://doi.org/10.1101/2021.02.11.21251571> | | |

| ***PART 2***  ***Standards of reporting*** | As a separate file, we require you to submit a completed **checklist** detailing how and where the matters detailed in the guideline are addressed in your paper. Do NOT submit the guideline itself. Indicate below what guideline you have used. [please note and use the appropriate extensions – eg. CONSORT extension for cluster trials] | **Checklist submitted[[1]](#footnote-2)**** |
| --- | --- | --- |
| Randomised (and quasi-randomised) controlled trial | CONSORT – Consolidated Standards of Reporting Trials |  |
| Qualitative studies | COREQ: Consolidated criteria for reporting qualitative research | X |
| Systematic Review of Controlled Trials | PRISMA - Preferred Reporting Items for Systematic Reviews and Meta-Analyses |  |
| Study of Diagnostic accuracy / assessment scale | STARD Standards for the Reporting of Diagnostic Accuracy studies |  |
| Observational cohort, case control and cross sectional studies | STROBE **St**rengthening the **R**eporting of **Ob**servational Studies in **E**pidemiology |  |
| Quasi experimental / non-randomized evaluations | TREND - Transparent Reporting of Evaluations with Non-randomized Designs |  |
| Other (please name / give source) | SRQR - Standards for Reporting Qualitative Research | X |
| Not applicable (please elaborate) | *If there is no applicable guideline, upload a blank file with the words ‘not applicable’ when requested at submission.* |  |

1. ^*^ [↑](#footnote-ref-2)
